# Myocardial Strain for the Differentiation of Myocardial Involvement in the Post-Acute Sequelae of COVID-19—A Multiparametric Cardiac MRI Study

**DOI:** 10.3390/tomography10030026

**Published:** 2024-02-27

**Authors:** El-Sayed H. Ibrahim, Jason Rubenstein, Antonio Sosa, Jadranka Stojanovska, Amy Pan, Paula North, Hallgeir Rui, Ivor Benjamin

**Affiliations:** 1 Department of Radiology, Medical College of Wisconsin, 8701 Watertown Plank Rd., Milwaukee, WI 53226, USA; asosa@mcw.edu; 2 Department of Medicine, Medical College of Wisconsin, 8701 Watertown Plank Rd., Milwaukee, WI 53226, USA; jrubenstein@mcw.edu (J.R.); ibenjamin@mcw.edu (I.B.); 3 Department of Radiology, New York University, 221 Lexington Ave, New York, NY 10016, USA; jadranka.stojanovska@nyulangone.org; 4 Department of Pediatrics, Medical College of Wisconsin, 8701 Watertown Plank Rd., Milwaukee, WI 53226, USA; apan@mcw.edu; 5 Department of Pathology, Medical College of Wisconsin, 8701 Watertown Plank Rd., Milwaukee, WI 53226, USA; pnorth@mcw.edu (P.N.); hrui@mcw.edu (H.R.)

**Keywords:** MRI, COVID-19, strain, strain rate, myocarditis

## Abstract

Myocardial involvement was shown to be associated with an unfavorable prognosis in patients with COVID-19, which could lead to fatal outcomes as in myocardial injury-induced arrhythmias and sudden cardiac death. We hypothesized that magnetic resonance imaging (MRI) myocardial strain parameters are sensitive markers for identifying subclinical cardiac dysfunction associated with myocardial involvement in the post-acute sequelae of COVID-19 (PASC). This study evaluated 115 subjects, including 65 consecutive COVID-19 patients, using MRI for the assessment of either post-COVID-19 myocarditis or other cardiomyopathies. Subjects were categorized, based on the results of the MRI exams, as having either ‘suspected’ or ‘excluded’ myocarditis. A control group of 50 matched individuals was studied. Along with parameters of global cardiac function, the MRI images were analyzed for measurements of the myocardial T1, T2, extracellular volume (ECV), strain, and strain rate. Based on the MRI late gadolinium enhancement and T1/T2/ECV mappings, myocarditis was suspected in 7 out of 22 patients referred due to concern of myocarditis and in 9 out of 43 patients referred due to concern of cardiomyopathies. The myocardial global longitudinal, circumferential, and radial strains and strain rates in the suspected myocarditis group were significantly smaller than those in the excluded myocarditis group, which in turn were significantly smaller than those in the control group. The results showed significant correlations between the strain, strain rate, and global cardiac function parameters. In conclusion, this study emphasizes the value of multiparametric MRI for differentiating patients with myocardial involvement in the PASC based on changes in the myocardial contractility pattern and tissue structure.

## 1. Introduction

To date, coronavirus disease 2019 (COVID-19), caused by the novel severe acute respiratory syndrome coronavirus-2 (SARS-CoV-2), has affected over 600 million people worldwide, of whom ~99% survived the pandemic. With nearly 1 million COVID-19 deaths in the United States and roughly one quarter of individuals infected with the virus experiencing some significant long-term sequelae, the spectrum of post-viral complications is only beginning to emerge. COVID-19 has now been established to be a multisystem disease, affecting different organs in the human body [1]. Especially, cardiac involvement has been shown to be associated with an unfavorable prognosis in patients with COVID-19, which could lead to fatal outcomes as in myocardial injury-induced arrhythmias and sudden cardiac death (SCD) [2,3]. Cardiac symptoms are increasingly recognized as late complications of COVID-19 infection in healthy individuals with mild initial illness [4,5]. Recent reports demonstrated the presence of cardiac sequelae not only in hospitalized patients with COVID-19 but also in outpatients, including elite athletes [6], as well as the persistence of cardiac complications beyond the acute stage and without any trend toward a decrease in these findings through the recovery period [7,8]. Nevertheless, the clinical significance of altered myocardial tissue composition and function in convalescing COVID-19 patients remains incompletely understood.

Two pathological mechanisms may be involved in post-COVID-19 myocardial involvement [9]. First, similar to SARS-CoV, SARS-CoV-2 uses the domain of the spike protein coding S for binding to the angiotensin-converting enzyme (ACE)-2 receptor on susceptible cells such as lung epithelial cells. Conceivably, the cardiotropism of SARS-CoV-2 for myocardial cells might cause viral myocarditis after infection. Second, indirect injury may be caused by an inflammatory storm induced by the immune response. Among 100 post-COVID-19 patients who were studied 2 to 3 months after the diagnosis, Puntmann et al. [10] reported cardiac involvement in 78%, with evidence of ongoing inflammation in 60%. This study suggested that COVID-19 might be responsible for a sustained subacute or chronic inflammatory state of the myocardium, comparable with cases of viral myocarditis and prone to cause long-term cardiac impairment by downstream activation of ventricular remodeling and fibrosis [11,12]. Therefore, inflammatory cardiac involvement and fibrosis development may constitute a pathophysiological common factor post-COVID-19, which is more pronounced in patients with persistent cardiac symptoms who are predisposed to a poor prognosis and the development of serious cardiac complications [10]. Among COVID-19-induced cardiac complications, myocarditis has clinical significance [13,14] because myocardial inflammation can result in permanent myocardial damage and contribute to the development of long-term cardiac sequelae, including arrhythmia, heart failure (HF), and SCD due to residual myocardial fibrosis or scarring [15,16]. This is similar to the case of chronic human immunodeficiency virus (HIV) infection, where postmortem heart biopsies have shown the presence of myocarditis in some HIV-infected patients [17].

Although endomyocardial biopsy is considered the gold standard for the diagnosis of myocarditis [18], it has the limitations of sampling error and limited sensitivity, and it carries inherent risks, including rare mortality [19]. Recently, MRI has emerged as an essential test of myocarditis because of its non-invasive nature, high sensitivity, and ability to comprehensively evaluate myocardial function, structure, and tissue characterization [20,21]. With a combination of different imaging sequences, the MRI-based Lake Louise Criteria (LLC) can reach an estimated area under the curve of 96% for the diagnosis of acute myocarditis in non-COVID-19 cohorts [20]. Specifically, the LLC require the existence of at least one T2-based criterion, e.g., a regional or global increase in the myocardial T2 relaxation time or an increased signal intensity in the T2-weighted MRI images, with at least one T1-based criterion, e.g., an increased myocardial T1, extracellular volume, or late gadolinium enhancement [20]. Although cardiac serum biomarkers, e.g., high-sensitivity cardiac troponin, are highly specific for myocardial injury, MRI has reported higher sensitivity for detecting occult cardiac involvement [9]. Besides generating conventional cardiac measures, e.g., ventricular ejection fraction (EF) and volumes, MRI is capable of myocardial tissue characterization using parametric mapping techniques, such as the T1, T2, and extracellular volume (ECV) mappings, and late gadolinium enhancement (LGE), which are sensitive markers for detecting diffuse fibrosis, inflammation, and edema [22,23]. Therefore, multiparametric MRI mapping may provide a sensitive tool for the non-invasive detection of the subset of patients who are at high risk of cardiac sequelae and more prone to myocardial damage in the PASC. Furthermore, given that patients with COVID-19-related myocarditis tend to have normal biventricular systolic function, MRI myocardial strain analysis could provide further insights into the subclinical cardiovascular sequelae after recovery from COVID-19 [24]. The subtle myocardial changes that might be detected by MRI strain imaging during the subclinical, potential reversible stages of the disease would be of a high clinical interest for the detection, prognostication, management, and follow-up of long COVID.

In this study, we hypothesize that the post-COVID-19 MRI strain and strain rate are sensitive biomarkers for the detection, description, and evaluation of the prevalence of subclinical cardiac dysfunction in patients with myocardial involvement in the PASC.

## 2. Materials and Methods

### 2.1. Study Design

**Figure 1:**
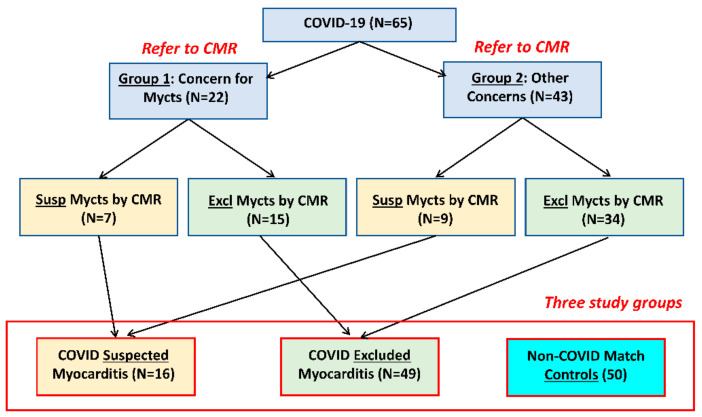
Study groups. A total of 65 consecutive COVID-19 patients who underwent MRI post-COVID-19 were included in the study. The patients were referred due to concerns of myocarditis (Mycts; group 1, n = 22) or other cardiac concerns (group 2, N = 43). MRI evaluation categorized the patients as either suspected or excluded myocarditis, which resulted in two study groups: ‘suspected myocarditis’ in 16 patients and ‘excluded myocarditis’ in 49 patients. We also included a third study group of non-COVID-19 matched controls (N = 50).

This study was approved by the Institutional Review Board. One hundred and fifteen subjects were included in this study (Figure 1, Table 1), including 65 consecutive COVID-19 patients (28 males, age = 47 ± 17 y.o.) who underwent MRI at our institution between September 2020 and February 2022 (duration between COVID-19 diagnosis and MRI = 159 ± 129 days) due to concern of post-COVID-19 myocarditis (group 1; n = 22) or other cardiomyopathies (group 2; n = 43) based on clinical examination, lab results of cardiac biomarkers, and symptoms. The main presentation symptoms were shortness of breath, chest pain, dyspnea, and palpitations. The endpoints were categorizing the subjects as either having ‘suspected’ or ‘excluded’ myocarditis based on the presence of non-ischemic LGE and/or elevated T1, ECV, and T2 values, as determined by the MRI LLC approach [20]. We also studied a control group of 50 subjects without COVID-19 infection and with age and sex distributions similar to those in the COVID-19 patients (24 males, age = 48 ± 15 y.o.), who underwent MRI at our institution due to concerns of different cardiovascular diseases with negative findings.

### 2.2. Clinical Presentation

The medical records of the study participants were reviewed to extract information about the risk factors, comorbidities, cardiac-related serum biomarkers (high-sensitivity cardiac troponin (hsTn), N-terminal pro b-type natriuretic peptide (NT proBNP), high-sensitivity c-reactive protein (hsCRP)), and COVID-19 vaccination, as available. Serum biomarkers collected on the day of the MRI scan were used in the analysis; otherwise, those collected on closest date to the MRI exam were used. A few outliers (hsTn > 50 ng/L, NT proBNP > 2000 pg/mL, hsCRP > 100 mg/L) were not included in the analysis in order to emphasize the measurement differences between the three study groups.

### 2.3. Image Acquisition

The COVID-19 patients underwent a standardized MRI protocol, including assessment of myocarditis based on changes in the myocardial tissue composition, as previously described [20]. The patients’ MRI images and exam reports were retrieved to obtain measures of global cardiac function, including the ventricular ejection fraction (EF) and indexed (divided by body surface area (BSA)) end-diastolic volume (EDV), end-systolic volume (ESV) and left ventricular (LV) mass. Multiparametric image analysis was also conducted to generate the myocardial strain and strain rate as well as T1, ECV, and T2 maps, as explained below. The MRI exams were conducted on Siemens 3T Skyra (49 COVID-19 patents and 26 controls) and 1.5T Aera (16 COVID-19 patients and 24 controls) scanners. The cine images were acquired using a balanced steady-state with free precession (bSSFP) sequence, where a stack of short-axis (SA) and long-axis (LA) images were acquired to cover the whole heart. A Modified Look-Locker inversion recovery (MOLLI) 5(3)3 sequence was used for acquiring pre- and post-contrast T1 mapping images during diastole. Three SA images were acquired at the basal, mid-ventricular, and apical levels. A T2-prepared bSSFP sequence was used for acquiring the T2 mapping images during diastole. Three SA images were acquired at the same locations as the T1 mapping images. The subjects also underwent MRI LGE imaging to determine the existence or absence of myocardial delayed enhancement. The subjects were injected with 0.2 mL/Kg of gadolinium contrast agent (Gadavist) and delayed enhancement images were acquired 10 min post-injection.

### 2.4. Image Analysis

The image analysis was conducted by one of the authors with 18 years of experience in MRI (E.I.) and validated by another author who is a board-certified cardiothoracic radiologist with 13 years of experience (A.S.). The existence of myocarditis was based on the revised Lake Luise Criteria (LLC) [20,21]. The reproducibility of the implemented analysis techniques has been previously reported [25]. The image analysis was conducted using the Circle cvi42 software version 5.13 (Circle, Calgary, AB, Canada). The generated parameters included the longitudinal, circumferential, and radial peak systolic strains (GLS, GCS, GRS) as well as the longitudinal, circumferential, and radial peak diastolic strain rates (GLSR, GCSR, GRSR). The longitudinal and circumferential strains and radial strain rate measurements were represented in absolute values (original values were negative) for clearer presentation. The endocardial and epicardial contours were identified on the T1 and T2 slices, where the cvi42 T1 and T2 mapping techniques were used to fit exponentially recovering (for T1) and decaying (for T2) curves at the pixel level to generate T1 and T2 maps for different slices.

### 2.5. Statistical Analysis

The statistical analysis was conducted on the generated data using the Prism GraphPad software version 9.3.1 (San Diego, CA, USA). The normality of the distributions were checked using the Shapiro–Wilk test. Continuous variables were represented as the mean ± standard deviation (SD), while categorical data were presented as the frequency and percentage. Analysis of variance (ANOVA) was conducted to examine the significance of the differences between different study groups (suspected myocarditis, excluded myocarditis, and controls), where *p* < 0.05 was considered statistically significant. The Welch correction was applied to correct for unequal variances. Statistical analysis of the T1 and T2 values was conducted separately for the 3T and 1.5T scans due to the effect of the magnetic field strength on the T1 and T2 values. Student’s *t*-test was conducted to investigate the differences in the strain and strain rate measurements between males vs. females, Black persons vs. White persons, and vaccinated vs. unvaccinated subjects within each of the study groups. Pearson’s correlation analysis was conducted to examine the associations between the different strains, strain rate parameters, and cardiac function parameters.

## 3. Results

### 3.1. Study Population

Table 1 shows a summary of the study population characteristics. Suspected myocarditis based on MRI (Figure 1) was raised in 16 patients (the ‘suspected myocarditis’ group, which included 7 patients from group 1 (COVID-19 patients referred for MRI due to concern of myocarditis) and 9 patients from group 2 (COVID-19 patients referred for MRI due to concern of other cardiac complications)), while myocarditis was excluded in the rest of the patients (the ‘excluded myocarditis’ group, which included 49 COVID-19 patients). It should be noted that the percentage of myocarditis could be affected by different factors, e.g., severity of COVID-19 or population bias.

The major risk factors were hypertension, hyperlipidemia, diabetes and obesity, while the major comorbidities were arrhythmias, cardiomyopathies, coronary artery disease (CAD) and HF, as shown in Table 1. The hsTn, NT proBNP, and/or hsCRP measurements, collected during follow-up visits, were available for 60 COVID-19 patients and for 14 control subjects (the reference normal values for these parameters were ≤10 ng/L, ≤449 pg/mL, and ≤3 mg/L, respectively, according to the analysis lab). Elevated biomarker measurements existed in the suspected myocarditis group, which were larger than those in the excluded myocarditis and control groups; however, the differences among the three groups were statistically insignificant (Table 2).

### 3.2. Global Cardiac Function Reflects Ventricular Remodeling Post-COVID-19 Infection

The global MRI parameters are presented in Table 2. The LVEF in the suspected myocarditis group was significantly lower than that in the excluded myocarditis group, which in turn was significantly lower than that in the control group. On the other hand, both the EDV and ESV in the suspected myocarditis group were higher than those in the excluded myocarditis group, which in turn were larger than those in the control group. Similarly, the LV mass in the suspected myocarditis group was larger than that in the excluded myocarditis and control groups, but the differences were not statistically significant. In the right ventricle (RV), the patterns of change in the EF and ESV were similar to those in the LV, although the differences were not statistically significant. However, the RV EDV measurements were similar in the three study groups.

### 3.3. Strain and Strain Rate Are Sensitive Markers for Identifying COVID-19 Patients with Myocarditis

**Figure 2:**
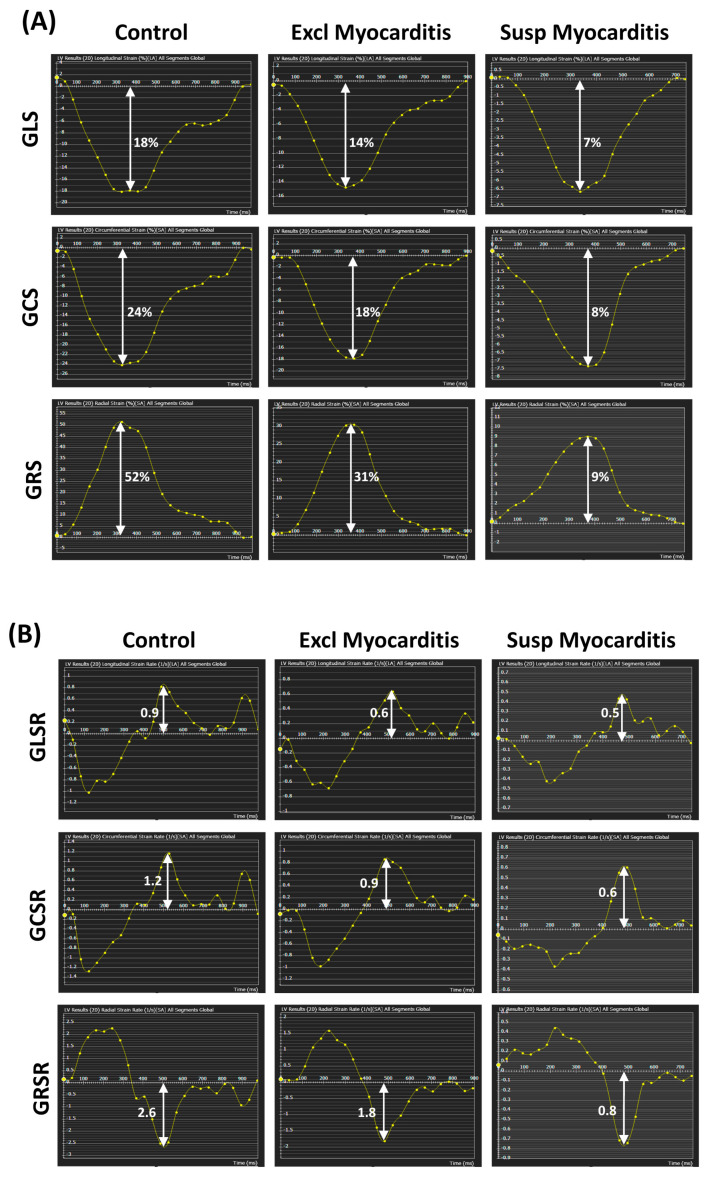
Myocardial strain and strain rate in the different study groups. Representative global (**A**) longitudinal (GLS), circumferential (GCS), and radial (GRS) strain curves. and global (**B**) longitudinal (GLSR), circumferential (GCSR), and radial (GRSR) strain rate curves for subjects in the control, excluded myocarditis, and suspected myocarditis groups. The vertical white arrows represent the peak systolic strains and peak diastolic strain rates. The peak systolic strains and peak diastolic strain rates (in absolute value) in the suspected myocarditis group are smaller than those in the excluding myocarditis group, which in turn are smaller than those in the control group (note that the vertical scale is different for each panel).

Examples of the myocardial strain and strain rate curves in the different study groups are shown in Figure 2. While the LGE pattern and ECV remodeling, indicating fibrosis, were the major factors for identifying suspected myocarditis, the global cardiac function measures (volumes, mass) did not show significant differences between the suspected and excluded myocarditis cases within each of the referral groups (groups 1 and 2). The LVEF showed only a significant difference between the suspected and excluded myocarditis in group 1, but not in group 2. However, the myocardial strain (in all directions) and strain rate (in the circumferential and radial directions) significantly differentiated between the suspected and excluded myocarditis inside groups 1 and 2, respectively.

**Figure 3:**
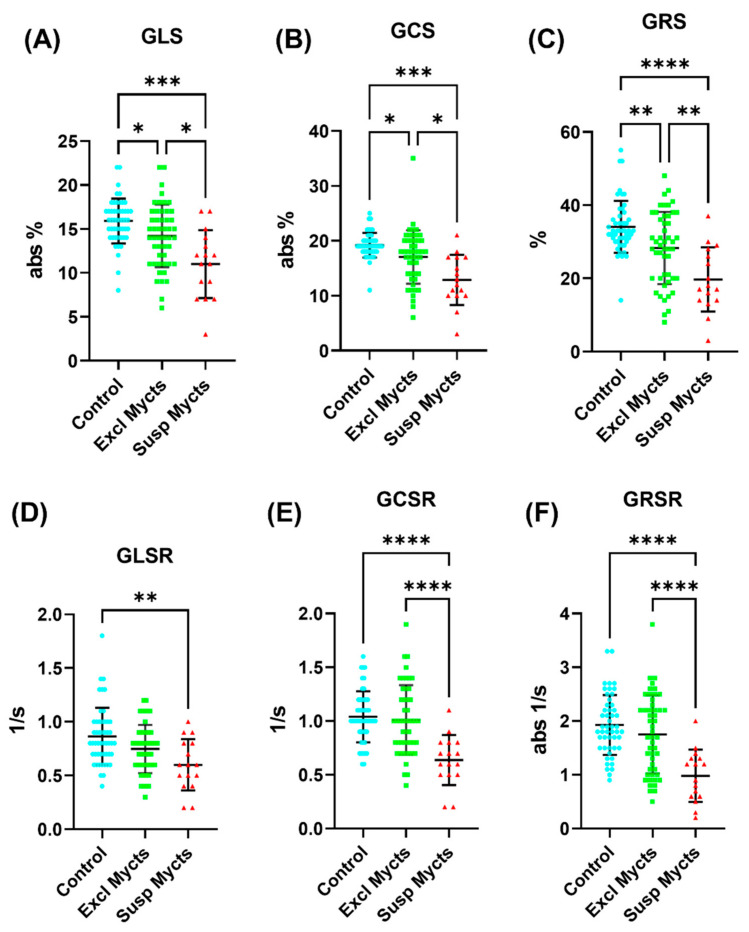
Global strain and strain rate analysis. Distribution of global (**A**) longitudinal (GLS), (**B**) circumferential (GCS) and (**C**) radial (GRS) strain measurements, and global (**D**) longitudinal (GLSR), (**E**) circumferential (GCSR) and (**F**) radial (GRSR) strain rate measurements in the control (blue), excluded myocarditis (green), and suspected myocarditis (red) groups. Note that the GLS, GCS, and GRSR are represented in absolute values (original measurements are negative) for presentation clarity. The vertical black lines mark the mean ± one standard deviation. Statistically significant (*p* < 0.05) differences between the study groups are represented by asterisks. The number of asterisks in the figure represent the significance level (number of zeros after decimal point in *p*).

In the group of all the MRI suspected myocarditis cases, the strain parameters were significantly lower than those in the group of all the MRI excluded myocarditis, as shown in Table 3 and Figure 3. The GLS and GRS in the suspected myocarditis group were significantly smaller than those in the excluded myocarditis group, which in turn were significantly smaller than those in the control group, respectively. On the regional level (base, mid-ventricle, apex), all the strain measurements, except for the apical longitudinal strain, showed significant reductions in the suspected myocarditis group compared to the control group, as shown in Supplementary Figure S1. Furthermore, all the strain measurements, except for the mid-ventricular and apical longitudinal strains, showed significant differences between the suspected myocarditis group and the excluded myocarditis group.

Regarding the strain rates (Table 3 and Figure 3), the GLSR, GCSR, and GRSR in the suspected myocarditis group were smaller than those in the excluded myocarditis group, which in turn were smaller than those in the control group (equal to it for the GCSR), respectively. All the strain rates showed significant differences between the suspected myocarditis group and the control group. Furthermore, the GCSR and GRSR showed significant differences between the suspected myocarditis and excluded myocarditis groups. On the regional level (base, mid-ventricle, apex), all the strain rates, except for the mid-ventricular and apical longitudinal strain rates, showed significant differences between the suspected myocarditis group and both the excluded myocarditis and control groups, as shown in Supplementary Figure S2.

Significant differences between males and females existed only in the GLS (13 ± 4% vs. 15 ± 3%, *p* = 0.035), GLSR (0.65 ± 0.24 s^−1^ vs. 0.81 ± 0.2 s^−1^, *p* = 0.017), and GRSR (1.46± 0.61 s^−1^ vs. 1.93 ± 0.74 s^−1^, *p* = 0.021) in the excluded myocarditis group, as well as in the GLS (15 ± 2% vs. 17 ± 3%, *p* = 0.010) in the control group, respectively. There existed significant differences between the White and Black COVID-19 patients in the GLS (14 ± 4% vs. 11 ± 3%, *p* < 0.001), GCS (17 ± 5% vs. 13 ± 4%, *p* = 0.007), GRS (28 ± 11% vs. 20 ± 7%, *p* = 0.005), GLSR (0.75 ± 0.23 s^−1^ vs. 0.59 ± 0.2 s^−1^, *p* = 0.016), GCSR (0.97 ± 0.37 s^−1^ vs. 0.74 ± 0.25 s^−1^, *p* = 0.012), and GRSR (1.68 ± 0.8 s^−1^ vs. 1.16 ± 0.47 s^−1^, *p* = 0.005), respectively. The differences in strains and strain rates between the COVID-19 vaccinated and unvaccinated subjects were statistically insignificant in all three groups, except for the GRS, GRSR, and GCSR in the excluded myocarditis group, where the COVID-19 vaccinated patients showed higher values than the unvaccinated patients, as follows: 32 ± 9% vs. 25 ± 10% (*p* = 0.012) for GRS, 2.1 ± 0.6 s^−1^ vs. 1.5 ± 0.7 s^−1^ (*p* = 0.004) for GRSR, and 1.1 ± 0.3 s^−1^ vs. 0.9 ± 0.3 s^−1^ (*p* = 0.013) for GCSR, respectively.

### 3.4. COVID-19 Affects Myocardial Tissue Structure

#### 3.4.1. LGE Findings

The LGE was a main finding in the suspected myocarditis patients. MRI revealed that 15 out of the 16 patients in the suspected myocarditis group had non-ischemic LGE patterns, mainly in the mid-myocardial and sub-epicardial regions. On the contrary, out of the 49 patients in the excluded myocarditis group, 7 patients had non-ischemic LGE patterns and 4 patients had ischemic LGE patterns. No subjects in the control group had LGE findings.

#### 3.4.2. ECV Measurements

**Figure 4:**
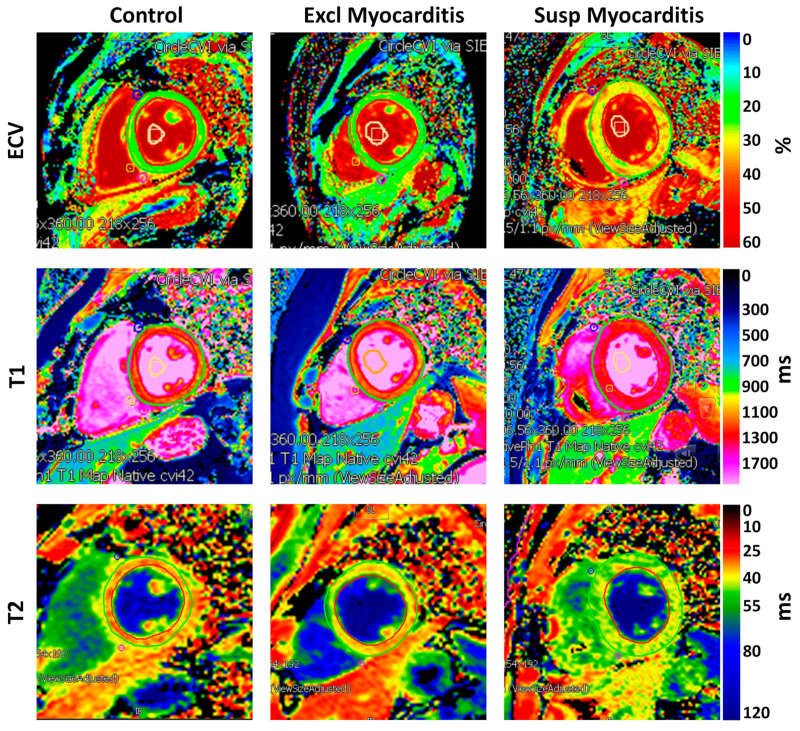
Myocardial parametric mapping. MRI-derived extracellular volume (ECV), T1, and T2 maps in subjects from the control, excluded myocarditis, and suspected myocarditis groups. All the parameters show larger values in the suspected myocarditis group compared to those in the excluded myocarditis group, which in turn are larger than those in the control group, reflecting increased diffuse fibrosis, inflammation, and edema.

**Figure 5:**
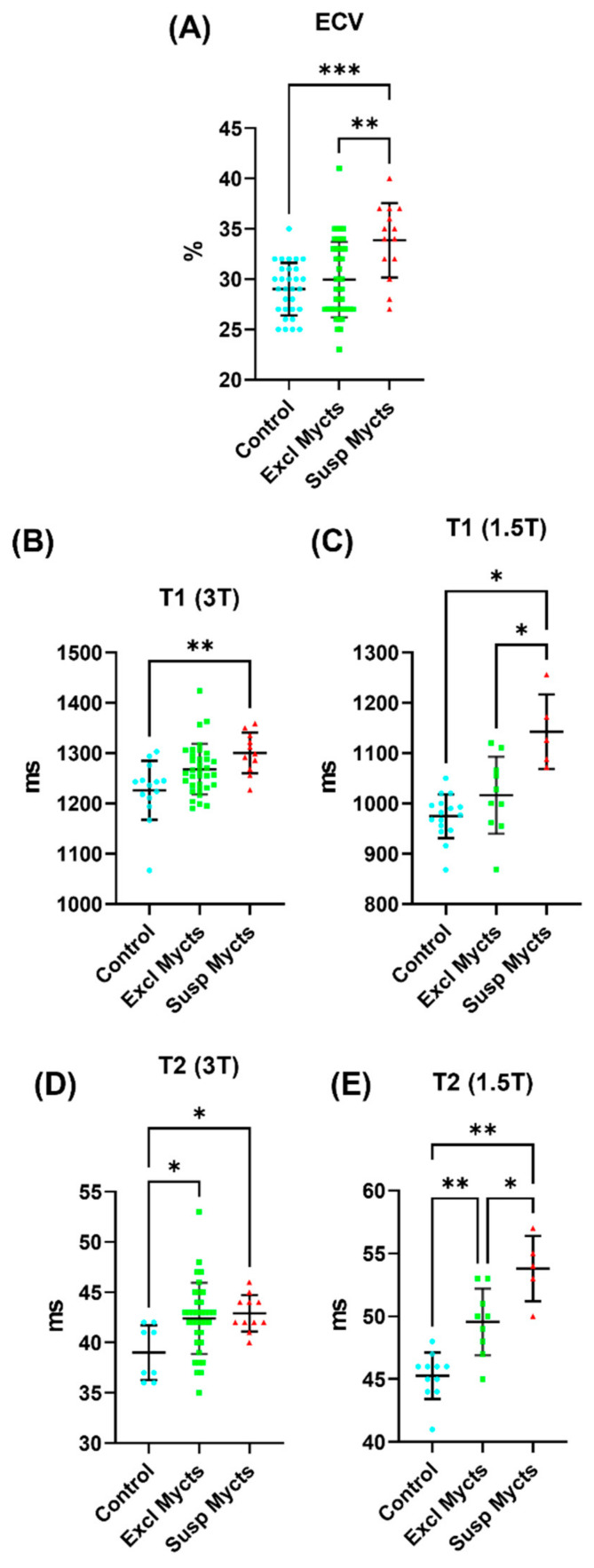
Global ECV, T1, and T2 analysis. (**A**) Extracellular volume (ECV) measurements in the control (blue), excluded myocarditis (green), and suspected myocarditis (red) groups. (**B**,**C**) Distribution of the T1 measurements from the (**B**) 3T scans and (**C**) 1.5T scans in the control (blue), excluded myocarditis (green), and suspected myocarditis (red) groups. (**D**,**E**) Distribution of the T2 measurements from the (**D**) 3T scans and (**E**) 1.5T scans in the control (blue), excluded myocarditis (green), and suspected myocarditis (red) groups. The vertical black lines show the mean ± one standard deviation. Statistically significant (*p* < 0.05) differences between the study groups are represented by asterisks. The number of asterisks in the figure represent the significance level (number of zeros after decimal point in *p*).

Figure 4 shows examples of the ECV maps and Table 4 summarizes the ECV measurements in the different study groups. In general, the COVID-19 patients, especially in the suspected myocarditis group, had higher MRI mapping values. The global ECV (Figure 5) in the suspected myocarditis group was larger than that in the excluded myocarditis group, which in turn was larger than that in the control group, where significant differences existed between the suspected myocarditis group and both the excluded myocarditis and control groups.

#### 3.4.3. T1 Measurements

Figure 4 shows examples of the MRI T1 maps and Table 4 summarizes the T1 measurements in the different study groups. The global native T1 measurements (Figure 5) in the 3T scans were 1301 ± 40 ms vs. 1268 ± 50 ms vs. 1226 ± 59 ms, and in the 1.5T scans were 1143 ± 74 ms vs. 1017 ± 77 ms vs. 975 ± 44 ms, in the suspected myocarditis vs. excluded myocarditis vs. control groups, respectively. The reference normal T1 values for the 3T and 1.5T scanners used in this study are <1300 ms and <1000 ms, respectively, as established at our institution. All the T1 measurements (calculated separately for 3T and 1.5T scans) in the suspected myocarditis group were larger than those in the excluded myocarditis group, which in turn were larger than those in the control group. The global T1 (Figure 5) showed significant differences between the suspected myocarditis group and the control group on the 3T scans, while it showed significant differences between the suspected myocarditis group and both the excluded myocarditis and control groups on the 1.5T scans.

#### 3.4.4. T2 Measurements

Figure 4 shows examples of the MRI T2 maps and Table 4 summarizes the T2 measurements in the different study groups. The global T2 measurements (Figure 5) in the 3T scans were 43 ± 2 ms vs. 42 ± 4 ms vs. 39 ± 3 ms, and in the 1.5T scans were 54 ± 3 ms vs. 50 ± 3 ms vs. 45 ± 2 ms, in the suspected myocarditis vs. excluded myocarditis vs. control groups, respectively. The reference normal T2 values for the 1.5T and 3T scanners used in this study are <50 ms and <40 ms, respectively, as established at our institution. Similar to the T1 measurements, all the global and regional T2 measurements (calculated separately for the 3T and 1.5T scans) in the suspected myocarditis group were larger than those in the excluded myocarditis group, which in turn were larger than those in the control group. The only exception was for the apical T2 measurement on the 3T scans between the suspected myocarditis (43 ± 4 ms) and excluded myocarditis (44 ± 4 ms) groups. Increased T2 values could be an indicator of tissue edema [20].

### 3.5. Associations between Cardiac Functional Parameters

**Figure 6:**
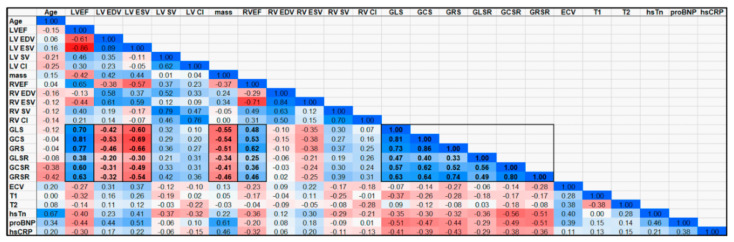
Correlation heatmap for all the studied parameters. The color scale represents the correlation values between −1 (red) and 1 (blue), while white represents zero correlation. Moderate-to-high positive and negative correlations are typed in BOLD font inside boxes. Specifically, there existed significant moderate correlations between the strain vs. strain rate parameters, and between both the strain and strain rate parameters vs. LVEF and RVEF (positive correlations) and vs. LVEDV, LVESV and LV mass (negative correlations).

**Figure 7:**
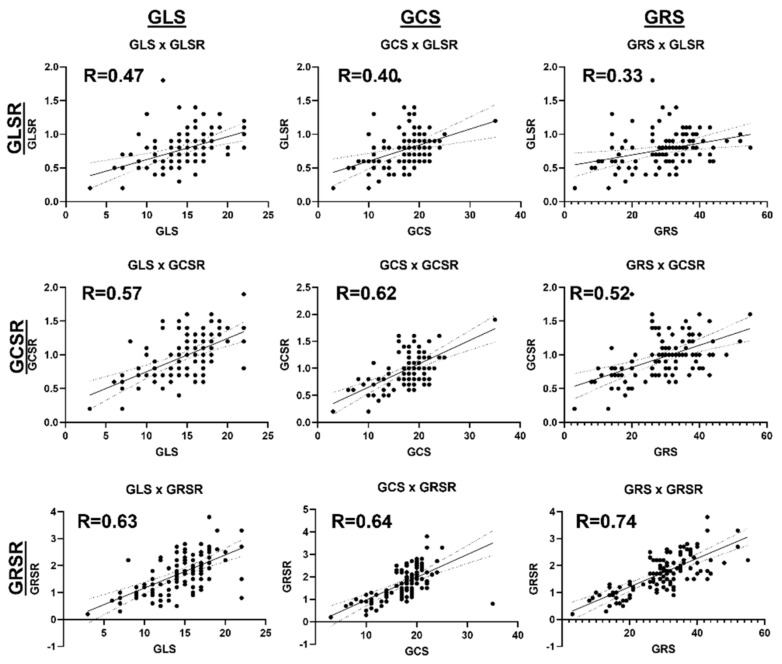
Linear regressions of the strain (GLS, GCS, GRS) vs. strain rate (GLSR, GCSR, GRSR) parameters. There exist significant moderate positive correlations between the strain and strain rate parameters in all directions, implying simultaneous deterioration in systolic and diastolic functions.

**Figure 8:**
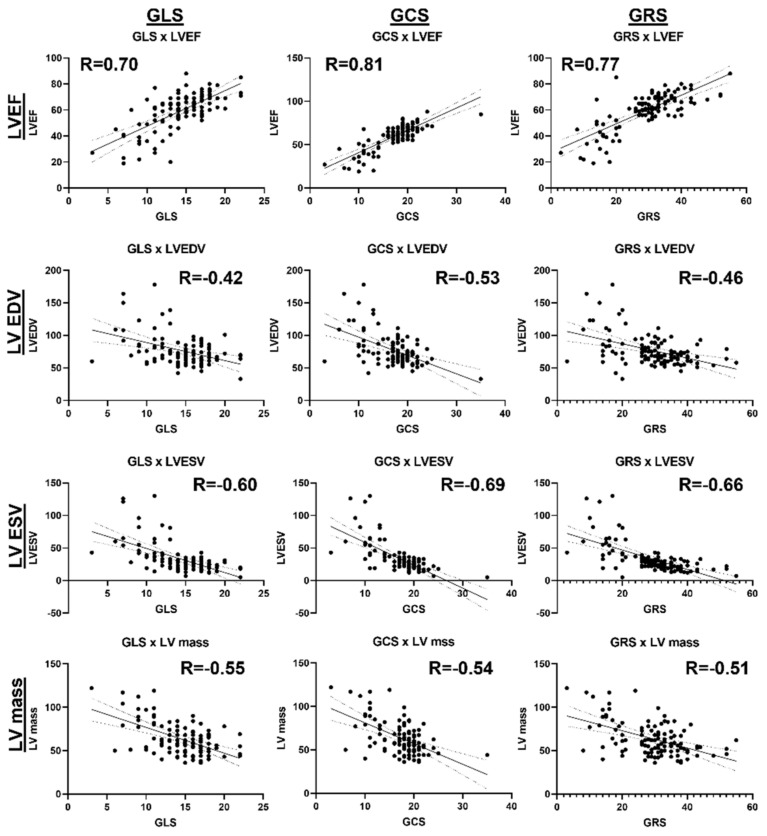
Linear regressions of the GLS, GCS, GRS strains vs. global cardiac function parameters. There exist significant moderate positive correlations between the strains/strain rates and LVEF, and significant moderate negative correlations between the strains/strain rates and LVEDV, LVESV, and LV mass, implying that deterioration in systolic function is accompanied by ventricular remodeling.

**Figure 9:**
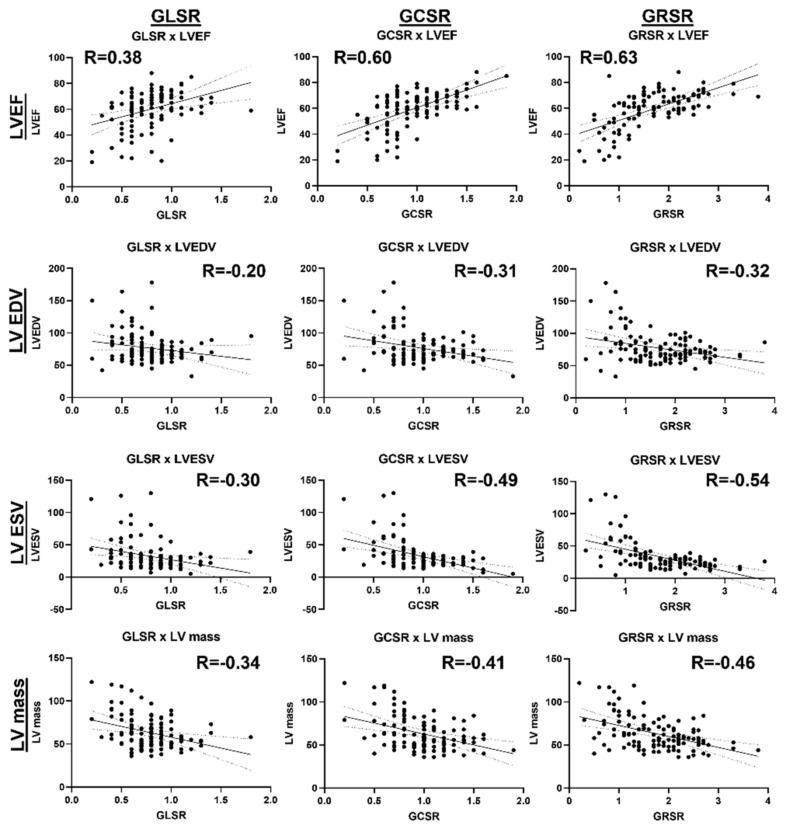
Linear regressions of the GLSR, GCSR, GRSR strain rates vs. global cardiac function parameters. There exist significant moderate positive correlations between the strains/strain rates and LVEF, and significant moderate negative correlations between the strains/strain rates and LVEDV, LVESV, and LV mass, implying that deterioration in systolic function is accompanied by ventricular remodeling.

Figure 6 shows a correlation heatmap between all the studied parameters. Significant moderate correlations are presented in BOLD font inside small boxes. In general, there existed significant moderate correlations between all the strain parameters (GLS, GCS, GRS) and all the strain rate parameters (GLSR, GCSR, GRSR), as shown in Figure 7. All the strain (Figure 8) and strain rate (Figure 9) parameters also showed significant moderate positive correlations with the LVEF and significant moderate negative correlations with the LV EDV, LV ESV, and LV mass.

## 4. Discussion

### 4.1. Study Findings and Strengths

In this study, we focused on a subgroup of COVID-19 patients with suspected myocarditis who are at higher risk of cardiac complications compared to other patients without myocardial involvement. The results demonstrated that patients with suspected myocarditis have significant increases in myocardial tissue characteristics (CV, T1, T2, LGE), which are accompanied by significant decreases in the tissue strain and strain rates, compared to patients without myocarditis. Using MRI to study COVID-19’s effects on altering the myocardial tissue structure and contractility performance (strain analysis) in this population has a great potential for future longitudinal studies. We have recently reported the potential profibrotic mechanism in COVID-19 by studying heat shock protein (HSP)-47+ myofibroblasts and CD163+ macrophages markers in explanted hearts from autopsied patients [26]. The results of the current study about the elevated T1 and ECV values in COVID-19 patients, reflecting increased diffuse myocardial fibrosis, suggest the possibility of these patients developing future fatal cardiac complications, including ventricular tachycardia and SCD. This is not unexpected considering the recently reported results about the role of fatal arrhythmias as a major cause of death in HIV, even more than that from coronary artery disease [17]. The results of this study also highlight the importance of myocardial strain analysis as a non-invasive means of the early detection of subclinical cardiac dysfunction in COVID-19 patients who develop myocarditis. Therefore, future studies that link non-invasive MRI parameters to profibrotic biomarkers from serum or biopsied tissues are warranted to establish the role of MRI in risk stratification and early detection of subclinical cardiac dysfunction in long COVID.

Another strength of this study is evaluating both systolic and diastolic heart functions using the peak systolic strain and peak diastolic strain rate, respectively. While it is important to assess myocardium contractility during systole, it is of equal importance to assess its relaxation pattern during diastole, where diastolic cardiac dysfunction typically precedes the development of HF with preserved EF (HFpEF), which accounts for up to 50% of HF cases [27]. The results showed significant moderate correlations between the strain and strain rate parameters, which demonstrated that COVID-19 results in simultaneous deterioration of both cardiac systolic and diastolic functions. LGE was found in almost all the suspected myocarditis subjects, who demonstrated the lowest strain and strain rate results. There were also significant moderate correlations between the strain/strain rate versus global cardiac function parameters, which demonstrated that myocardial contractility deterioration occurs during an ongoing process of ventricular remodeling. This study shed the light on the limitations of conventional serum cardiac biomarkers, as there existed insignificant differences in these parameters among the three studied groups, and several subjects who showed elevated biomarkers had myocarditis excluded by MRI. However, the results demonstrated no associations between the MRI parameters and the patients’ age or duration between COVID-19 diagnosis and MRI exam, suggesting that cardiac complications in the PASC may occur in young healthy subjects and may persist for a long time post-infection.

The results demonstrated differences in the global (EF and mass) and regional (strain, strain rate) cardiac function parameters between White and Black patients, where White persons had better cardiac function than Black persons. Such differences could be attributed to genetic differences between the two races that put Black persons at a higher risk of cardiac complications post-COVID-19 infection [28,29].

### 4.2. Limitations

This study has some limitations. First, this is a single-center, retrospective observational study on 115 subjects (65 COVID-19 patients and 50 matched controls); therefore, the generalizability of our findings may be limited and the need for longitudinal follow-up studies is clear. Furthermore, some lab results and/or images were not available or had some artifacts, which is not unexpected due to the retrospective nature of the study. Also, the relatively long period of time between the COVID-19 disease and MRI exam may have allowed for some missing events that were not caught during this period. However, based on the promising results of this study, we are planning to conduct a follow-up prospective longitudinal study on a larger cohort to assess the progression of cardiac complications in long COVID. Another limitation of this study is that we had no previous MRI for comparison with the post-COVID-19 exams. Furthermore, we did not have access to details about the COVID-19 symptoms, course, or used medications. Nevertheless, this should not affect the study results, which focused on the role of MRI in evaluating cardiac complications in the PASC, especially in high-risk patients with myocardial involvement regardless of the experienced COVID-19 symptoms.

Another limitation of this study is that the MRI scans were conducted on two scanners (1.5T and 3T). However, the same imaging protocol/sequences were used on both scanners. It should be also noted that the generated quantitative MRI measures, except for the T1 and T2, are not affected by the magnetic field strength. Therefore, separate analyses (for 1.5T scans and 3T scans) were conducted on the T1 and T2 parameters, where the results of both analyses led to the same conclusions about the patterns of change in these parameters among the different studied groups. Finally, the results of the MRI could not be confirmed by histopathological analysis [18], although there are controversies and limitations about the use of myocardial biopsy, as explained earlier in the paper [19]. However, the value of MRI mappings for assessing diffuse fibrosis, inflammation, and edema has already been established in the literature [14,20,30,31], which extends the potential application of the results of this study to the more general case of myocardial involvement in the PASC, including the underlying pathways of myocardial inflammation and fibrosis development, as suggested by other studies [9,10,12].

Finally, it should be noted that speckle-tracking echocardiography could be used for strain analysis in case MRI data are not available. Another note is that other factors besides myocarditis could lead to decreased strain measurements [32] and that the high prevalence of myocarditis has been reported in other studies, e.g., [33]. Finally, the difference in the time between COVID-19 and MRI between the myocarditis and non-myocarditis groups may have a biasing effect on the results that should be investigated in a larger study along with other influencing factors, e.g., age, sex, and comorbidities.

## 5. Conclusions

In conclusion, this study emphasizes the value of multiparametric MRI for differentiating patients at risk of myocardial involvement in the PASC based on changes in the myocardial contractility pattern and tissue characteristics. Especially, the strain and strain rates are shown to be sensitive markers that can detect subclinical cardiac dysfunction. Because of the long-term nature of the post-acute sequelae of COVID-19, there may be long-lasting consequences for patients and health systems; therefore, future prospective longitudinal studies on large cohorts are warranted.

## Figures and Tables

**Table 1:** Study population demographics, risk factors and comorbidities.

Parameter (Unit)	Control	Excluded Myocarditis	Suspected Myocarditis
Number of subjects	50	49	16
Sex (M/F)	24/26	19/30	9/7
Age (years; mean ± SD)	48 ± 15	44 ± 17	54 ± 15
BSA (m^2^; mean ± SD)	2.06 ± 0.29	2.11 ± 0.32	2.16 ± 0.31
Heart rate (bpm; mean ± SD)	64 ± 12	66 ± 13	74 ± 15
Days between COVID diagnosis and MRI (mean ± SD)	--	176 ± 130	98 ± 106
COVID vaccinated	18 (36%)	20 (41%)	3 (19%)
White persons	45 (90%)	37 (76%)	10 (63%)
Black persons	5 (10%)	9 (18%)	5 (31%)
Other	0 (0%)	3 (6%)	1 (6%)
Non-Hispanic	50 (100%)	47 (96%)	15 (94%)
Hispanic	0 (0%)	2 (4%)	1 (6%)
Hypertension	18 (36%)	7 (14%)	9 (56%)
Hyperlipidemia	15 (30%)	5 (10%)	4 (25%)
Diabetes	6 (12%)	3 (6%)	6 (38%)
Obesity	7 (14%)	7 (14%)	4 (25%)
Arrhythmias	17 (34%)	7 (14%)	4 (25%)
Cardiomyopathy	3 (6%)	2 (4%)	5 (31%)
CAD	1 (2%)	5 (10%)	5 (31%)
HF	10 (20%)	7 (14%)	11 (69%)

Abbreviations: BSA: body surface area; CAD: coronary artery disease; HF: heart failure.

**Table 2:** Serum cardiac biomarkers and MRI global cardiac function parameters (mean ± SD).

Parameter (Unit)	Control	Excluded Myocarditis	P1	Suspected Myocarditis	P2/P3
hsTn (ng/L)	11.4 ± 6.6	11.9 ± 11.2	0.998	28.2 ± 20	0.101/0.117
NT proBNP (pg/mL)	187 ± 208	384 ± 452	0.336	627 ± 583	0.313/0.722
hsCRP (mg/L)	8.4 ± 9.5	4.4 ± 5.2	0.744	9.8 ± 9.7	0.991/0.343
LV EF (%) ^#^*^†^	67 ± 7	58 ± 13	<0.001	44 ± 17	<0.001/0.014
LV EDV (ml/m^2^)	70 ± 12	78 ± 24	0.073	93 ± 37	0.070/0.382
LV ESV (ml/m^2^) ^#^*	23 ± 7	35 ± 21	0.001	56 ± 36	0.008/0.108
LV CI (L/min/m^2^)	2.9 ± 0.7	2.8 ± 0.8	0.672	2.7 ± 0.8	0.567/0.942
LV Mass (g/m^2^)	61 ± 13	62 ± 19	0.975	78 ± 28	0.079/0.124
RV EF (%) *	56 ± 7	53 ± 10	0.113	46 ± 14	0.032/0.230
RV EDV (ml/m^2^)	79 ± 16	77 ± 20	0.942	78 ± 30	>0.999/0.998
RV ESV (ml/m^2^)	35 ± 10	37 ± 14	0.709	44 ± 29	0.486/0.704
RV CI (L/min/m^2^)	2.8 ± 0.7	2.6 ± 0.8	0.346	2.6 ± 1.1	0.810/>0.999

P1 (^#^), P2 (*), and P3 (^†^) represent statistical significance of control vs. excluded myocarditis, control vs. suspected myocarditis, and suspected myocarditis vs. excluded myocarditis, respectively. Abbreviations: hsTn: high-sensitivity cardiac troponin; NT proBNP: N-terminal brain natriuretic peptide; hsCRP: high-sensitivity c-reactive protein; EF: ejection fraction; EDV: indexed end-diastolic volume; ESV: indexed end-systolic volume; CI: cardiac index.

**Table 3:** MRI regional cardiac function parameters (mean ± SD).

Parameter (Unit)	Control	Excluded Myocarditis	P1	Suspected Myocarditis	P2/P3
GLS (abs %) ^#^*^†^	16 ± 3	14 ± 4	0.023	11 ± 4	<0.001/0.021
LS-B (abs %) *^†^	20 ± 4	19 ± 5	0.376	14 ± 4	<0.001/<0.001
LS-M (abs %) ^#^*	14 ± 3	12 ± 4	0.013	11 ± 4	0.021/0.661
LS-A (abs %)	14 ± 4	13 ± 4	0.641	11 ± 6	0.111/0.298
GCS (abs %) ^#^*^†^	19 ± 2	17 ± 5	0.019	13 ± 5	<0.001/0.013
CS-B (abs %) *^†^	18 ± 3	17 ± 5	0.301	12 ± 4	<0.001/0.007
CS-M (abs %) ^#^*^†^	19 ± 3	16 ± 5	0.010	12 ± 5	0.001/0.035
CS-A (abs %) *^†^	24 ± 3	22 ± 7	0.120	17 ± 6	<0.001/0.033
GRS (%) ^#^*^†^	34 ± 7	28 ± 10	0.004	20 ± 9	<0.001/0.008
RS-B (%) ^#^*^†^	32 ± 7	28 ± 9	0.033	20 ± 9	<0.001/0.009
RS-M (%) ^#^*^†^	32 ± 8	26 ± 9	0.002	19 ± 10	<0.001/0.044
RS-A (%) ^#^*^†^	51 ± 13	42 ± 18	0.020	30 ± 14	<0.001/0.024
GLSR (1/s) *	0.9 ± 0.27	0.7 ± 0.22	0.058	0.6 ± 0.24	0.003/0.114
LSR-B (1/s) *^†^	1.3 ± 0.43	1.2 ± 0.41	0.294	0.9 ± 0.28	<0.001/0.017
LSR-M (1/s)	0.9 ± 0.27	0.8 ± 0.28	0.986	0.7 ± 0.29	0.074/0.117
LSR-A (1/s)	0.9 ± 0.33	0.9 ± 0.31	>0.999	0.8 ± 0.35	0.165/0.171
GCSR (1/s) *^†^	1.0 ± 0.24	1.0 ± 0.32	0.937	0.6 ± 0.23	<0.001/<0.001
CSR-B (1/s) *^†^	1.1 ± 0.27	1.0 ± 0.36	0.649	0.7 ± 0.29	<0.001/<0.001
CSR-M (1/s) *^†^	1.1 ± 0.26	1.0 ± 0.35	0.629	0.6 ± 0.27	<0.001/<0.001
CSR-A (1/s) *^†^	1.5 ± 0.41	1.5 ± 0.50	0.881	1.1 ± 0.43	<0.002/0.012
GRSR(abs 1/s) *^†^	1.9 ± 0.56	1.8 ± 0.73	0.441	1.0 ± 0.49	<0.001/<0.001
RSR-B (abs 1/s) *^†^	2.1 ± 0.63	1.8 ± 0.73	0.092	1.1 ± 0.64	<0.001/0.002
RSR-M (abs 1/s) *^†^	1.8 ± 0.46	1.6 ± 0.64	0.290	0.9 ± 0.46	<0.001/<0.001
RSR-A (abs 1/s) *^†^	3.3 ± 1.11	3.0 ± 1.54	0.607	1.9 ± 1.07	<0.001/0.006

Almost all the strain and strain rate measurements showed significant (*p* < 0.05) reductions in suspected myocarditis vs. excluded myocarditis and/or control subjects. P1 (^#^), P2 (*), and P3 (^†^) represent the statistical significance of control vs. excluded myocarditis, control vs. suspected myocarditis, and suspected myocarditis vs. excluded myocarditis, respectively. Abbreviations: GLS, GCS and GRS: global longitudinal, circumferential and radial strains; GLSR, GCSR and GRSR: global longitudinal, circumferential and radial strain rates; LS, CS and RS: longitudinal, circumferential and radial strains; LSR, CSR and RSR: longitudinal, circumferential and radial strain rates; B, M and A: basal, mid-ventricular and apical.

**Table 4:** MRI myocardial tissue characteristics (mean ± SD).

Parameter (Unit)	Controls	Excluded Myocarditis	P1	Suspected Myocarditis	P2/P3
LGE (subjects)	0	11	--	15	--
ECV-G (%) *^†^	29 ± 3	30 ± 4	0.486	34 ± 4	<0.001/0.007
ECV-B (%) *^†^	29 ± 3	28 ± 4	0.934	34 ± 4	<0.001/<0.001
ECV-M (%) *^†^	28 ± 3	29 ± 3	0.979	33 ± 4	0.003/0.005
ECV-A (%) *	30 ± 3	32 ± 8	0.247	35 ± 5	0.006/0.312
T1_3T-G (ms) *	1226 ± 59	1268 ± 50	0.081	1301 ± 40	0.003/0.118
T1_3T-B (ms) *	1228 ± 56	1266 ± 63	0.126	1301 ± 40	0.003/0.108
T1_3T-M (ms) *	1218 ± 59	1261 ± 53	0.075	1299 ± 50	0.004/0.134
T1_3T-A (ms)	1236 ± 70	1293 ± 80	0.075	1301 ± 53	0.050/0.969
T1_1.5T-G (ms) *^†^	975 ± 44	1017 ± 77	0.346	1143 ± 74	0.013/0.042
T1_1.5T-B (ms)	1005 ± 35	1039 ± 58	0.307	1199 ± 128	0.070/0.111
T1_1.5T-M (ms) *	983 ± 58	1006 ± 89	0.842	1116 ± 78	0.045/0.102
T1_1.5T-A (ms) *	936 ± 64	1011 ± 103	0.160	1079 ± 41	<0.001/0.241
T2_3T-G (ms) ^#^*	39 ± 3	42 ± 4	0.030	43 ± 2	0.014/0.896
T2_3T-B (ms) ^#^*	38 ± 2	42 ± 4	0.008	43 ± 3	0.007/0.801
T2_3T-M (ms) *	39 ± 3	42 ± 4	0.078	43 ± 2	0.038/0.833
T2_3T-A (ms)	41 ± 4	44 ± 4	0.186	43 ± 4	0.402/0.970
T2_1.5T-G (ms) ^#^*^†^	45 ± 2	50 ± 3	0.003	54 ± 3	0.002/0.048
T2_1.5T-B (ms) ^#^*^†^	44 ± 1	48 ± 3	0.012	53 ± 2	0.001/0.015
T2_1.5T-M (ms) ^#^*^†^	46 ± 1	50 ± 3	0.003	53 ± 2	<0.001/0.033
T2_1.5T-A (ms) ^#^*	47 ± 3	51 ± 3	0.025	55 ± 4	0.025/0.235

P1 (^#^), P2 (*), and P3 (^†^) represent statistical significance of the control vs. excluded myocarditis, control vs. suspected myocarditis, and suspected myocarditis vs. excluded myocarditis, respectively. Abbreviations: LGE: late gadolinium enhancement; G, B, M and A: global, basal, mid-ventricular and apical; ECV: extracellular volume; T1_3T: myocardial T1 measurements from 3T MRI scans;T1_1.5T: myocardial T1 measurements from 1.5T MRI scans; T2_3T: myocardial T2 measurements from 3T MRI scans; T2_1.5T: myocardial T2 measurements from 1.5T MRI scans.

## Data Availability

Study data are available upon request from the corresponding author.

## Supplementary Materials

The following supporting information can be downloaded at https://www.mdpi.com/article/10.3390/tomography10030026/s1, Figure S1: Regional strain analysis; Figure S2: Regional strain rate analysis.
