# Supplementary material for: Myocardial Strain for the Differentiation of Myocardial Involvement in the Post-Acute Sequelae of COVID-19—A Multiparametric Cardiac MRI Study
Source: Tomography. 2024 Feb 27;10(3):331–48. doi: 10.3390/tomography10030026 (PMC10974260; doi:10.3390/tomography10030026)
Supplement: Supplementary file 1 [file tomography-10-00026-s001.zip › tomography-2748583-supplementary.pdf]

# Supplementary Materials

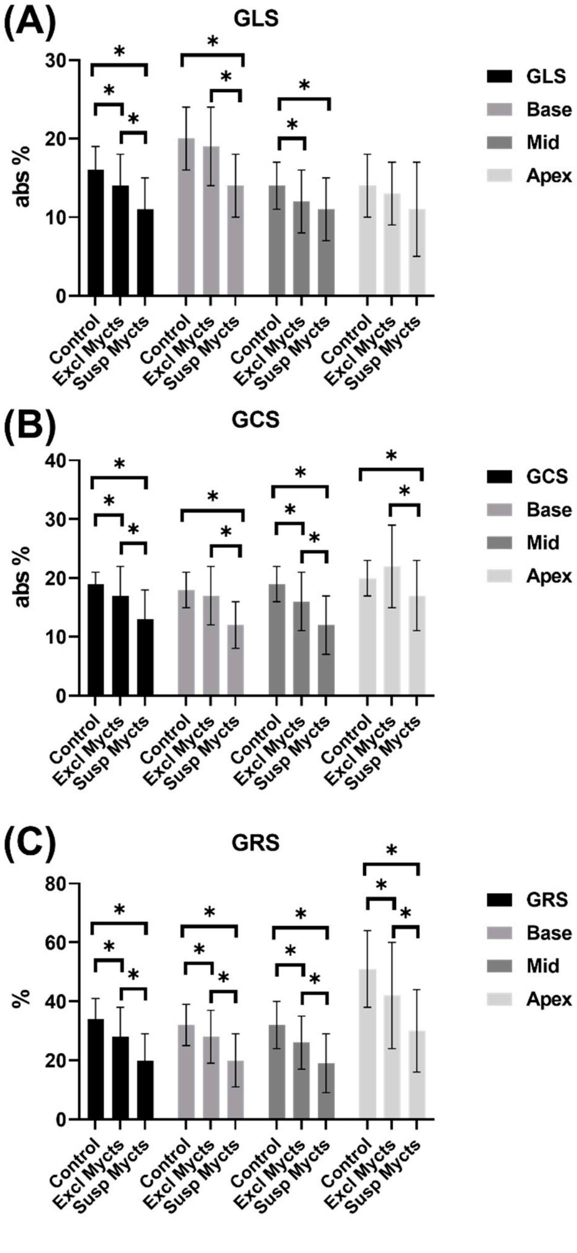


**Figure S1** Regional strain analysis. Global and regional (basal, mid-ventricular, and apical) strain results in the (A) longitudinal (GLS), (B) circumferential (GCS), and (C) radial (GRS) directions. The vertical black lines mark the mean ± one standard deviation. Almost all the measurements show smaller mean strain values in the suspected myocarditis (Myct) group compared to those in the excluded myocarditis group, which in turn are smaller than those in the control group. Most of the differences between the different study groups are statistically significant (*). Note that GLS and GCS are represented by absolute values (original values are negative).

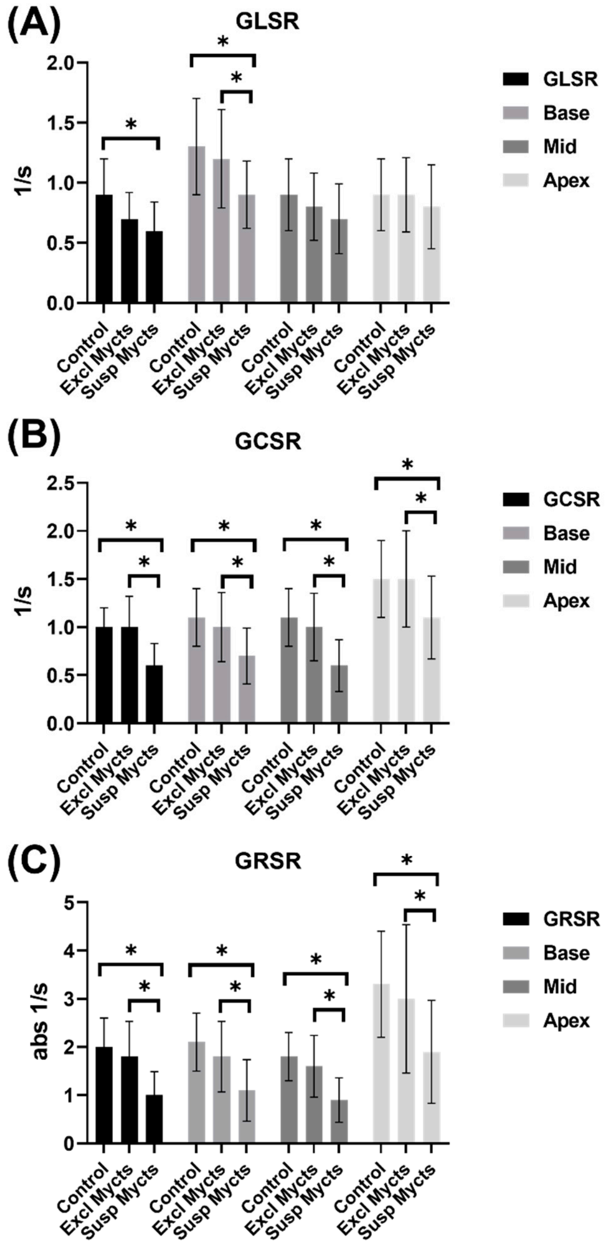


**Figure S2.** Regional strain rate analysis. Global and regional (basal, mid-ventricular, and apical) strain rate results in the (A) longitudinal (GLSR), (B) circumferential (GCSR), and (C) radial (GRSR) directions. The vertical black lines mark the mean ± one standard deviation. Almost all the measurements show smaller mean strain rate values in the suspected myocarditis (Myects) group compared to those in the excluded myocarditis group, which in turn are smaller than those in the control group. Most of the differences between the different study groups are statistically significant (*), especially for GCSR and GRSR. Note that GRSR is represented by absolute values (original values are negative).
